# The effects of interventions targeting multiple health behaviors on smoking cessation outcomes: a rapid realist review protocol

**DOI:** 10.1186/s13643-018-0702-0

**Published:** 2018-03-01

**Authors:** Nadia Minian, Wayne K. deRuiter, Mathangee Lingam, Tricia Corrin, Rosa Dragonetti, Heather Manson, Valerie H. Taylor, Laurie Zawertailo, Arezoo Ebnahmady, Osnat C. Melamed, Terri Rodak, Margaret Hahn, Peter Selby

**Affiliations:** 1 Centre for Addiction and Mental Health, 100 Stokes Street, Toronto, Ontario Canada; 2 Women’s College Hospital, 76 Grenville Street, Toronto, Ontario Canada; 3 Public Health Ontario, 480 University Avenue, Toronto, Ontario Canada; 4 Department of Family and Community Medicine, University of Toronto, 500 University Ave, Toronto, Ontario Canada; 5 Psychiatry, University of Toronto, 250 College Street 8th Floor, Toronto, Ontario Canada; 6 Dalla Lana School of Medicine, University of Toronto, 155 College St, Toronto, Ontario Canada; 7 Institute of Medical Sciences, 1 King’s College Circle, Toronto, Ontario Canada; 8 Pharmacology & Toxicology, University of Toronto, 1 King’s College Circle, Toronto, Ontario Canada

**Keywords:** Multiple health behaviors, Protocol, Realist review, Knowledge synthesis, Smoking cessation

## Abstract

**Background:**

Health behaviors directly impact the health of individuals, and populations. Since individuals tend to engage in multiple unhealthy behaviors such as smoking, excessive alcohol use, physical inactivity, and eating an unhealthy diet simultaneously, many large community-based interventions have been implemented to reduce the burden of disease through the modification of multiple health behaviors. Smoking cessation can be particularly challenging as the odds of becoming dependent on nicotine increase with every unhealthy behavior a smoker exhibits. This paper presents a protocol for a rapid realist review which aims to identify factors associated with effectively changing tobacco use and target two or more additional unhealthy behaviors.

**Methods:**

An electronic literature search will be conducted using the following bibliographic databases: MEDLINE, Embase, PsycINFO, Cumulative Index to Nursing and Allied Health Literature (CINAHL), The Cochrane Library, Social Science Abstracts, Social Work Abstracts, and Web of Science. Two reviewers will screen titles and abstracts for relevant research, and the selected full papers will be used to extract data and assess the quality of evidence. Throughout this process, the rapid realist approach proposed by Saul et al., 2013 will be used to refine our initial program theory and identify contextual factors and mechanisms that are associated with successful multiple health behavior change.

**Discussion:**

This review will provide evidence-based research on the context and mechanisms that may drive the success or failure of interventions designed to support multiple health behavior change. This information will be used to guide curriculum and program development for a government funded project on improving smoking cessation by addressing multiple health behaviors in people in Canada.

**Systematic review registration:**

PROSPERO CRD42017064430

**Electronic supplementary material:**

The online version of this article (10.1186/s13643-018-0702-0) contains supplementary material, which is available to authorized users.

## Background

Major chronic diseases, including cardiovascular diseases (CVDs), cancer, chronic respiratory diseases and diabetes, are the cause of 65% of all deaths in Canada each year [1]. Non-compliance with existing guidelines for tobacco use, physical activity, nutrition, and alcohol consumption account for nearly 50% of deaths and a life expectancy that is 17.9 years shorter compared to Canadians that practice healthy behaviors [2] and impose substantial and unnecessary economic strain to the healthcare system. In 2013, it was estimated that $9.6 billion in healthcare costs were attributable to newly diagnosed cancers that were preventable had Canadians adhered to recommendations for various health behaviors [3]. Considering that 22–93% of the Canadian population participate in multiple unhealthy behaviors [4–6], it is essential to continue to develop and implement large-scale population based interventions that focus on changing multiple health behaviors.

Tobacco use has a substantial impact on life expectancy, especially across specific demographic groups [2]. Therefore, interventions that are designed to focus on adopting and maintaining smoking cessation could be advantageous. Evidence suggests that unhealthy behaviors tend to cluster with tobacco use [7–9]. Engaging in these additional unhealthy behaviors can influence tobacco use as well as nicotine dependence [10, 11]. Thus, smoking cessation can prove to be increasingly challenging as the odds of becoming dependent upon nicotine increase by 23% for every unhealthy behavior a smoker exhibits [10]. Furthermore, studies have shown that reductions in tobacco use are significantly associated with participating in physical activity and lowering alcohol consumption [12–15]. For these reasons, interventions that attempt to change multiple health behaviors concurrent with smoking cessation could be beneficial.

Large community-based programs and interventions including the Multiple Risk Factor Intervention Trial (MRFIT), North Karelia Project, Pawtucket Heart Health Program, Stanford Five-City Project, Minnesota Heart Health Program, and the Mediterranean Lifestyle Program were developed and implemented to decrease the risk of coronary heart disease (CHD) mortality through the simultaneous reduction of multiple health behaviors such as tobacco use, unhealthy diet, and a lack of physical activity [16–22]. More recently, Project PREVENT was designed to reduce behavioral risk factors for colorectal cancer and the BETTER Trial aimed to improve the primary prevention of chronic diseases and the lifestyle factors associated with these diseases [16, 23].

Based upon theories for behavior change, these comprehensive interventions involved changing multiple behaviors through community-based interventions and health education strategies. Programs and interventions are often complex, and the outcomes can vary depending on individual, social, and environmental factors such as location, culture, available resources and population profile. Often, multi-level contextual factors and mechanisms pertaining to the development and implementation of successful behavioral change interventions are not fully understood. A realist review aims to narrow this gap by using a theory-based explanatory approach to explain complex interventions and to attempt to understand for whom, how, and under which circumstances the interventions are successful [24].

The purpose of this rapid realist review is to identify the factors associated with effectively reducing tobacco use as well as two or more additional unhealthy behaviors. We chose to evaluate interventions that endeavor to change three behaviors or more based on previous large-scale population-based interventions [16, 17, 25]. The information gathered from this realist review will provide evidence-based research to guide the curriculum and program development for *Picking up the PACE (Promoting and Accelerating Change through Empowerment): Expanding Tobacco Cessation Training Curriculum to Promote Integrated Chronic Disease Prevention*, a recently funded project by PHAC (PHAC grant #1617-HQ-000045). Developed with our expert panel, our specific research question is ‘What factors are associated with effective multiple health behavior change (three or more, including smoking)?’ In this protocol, we outline our initial program theory and describe the specific steps that will be undertaken to answer this question.

### Realist synthesis

Realist synthesis has its roots in realism, a philosophy situated between positivism (the belief that there is a real world out there that can be observed) and constructivism (the belief that reality is socially constructed and thus cannot be observed). Realists believe that reality can be stratified in events that can be observed, as well as the un-observable, and the context specific mechanisms behind them. Thus, when using a realist approach for evaluations, or synthesis, there is an assumption that there are many dimensions and layers that could explain any complex intervention. A realist review does not seek to explain all these layers; instead, it focuses on patterns between contexts and mechanisms, which create preconditions for particular behavioral outcomes [26].

The goal of realist synthesis is to understand how complex interventions work, or why they fail, in specific contexts and settings. ‘Theories’ rather than ‘programs’ are the unit of analysis [24]. Realist reviews propose that this is best accomplished by understanding the context-mechanism-outcome (C-M-O configurations) [24]. Context refers to social, cultural, historical, and institutional norms in which the program is introduced [27]. Mechanisms can be described as “agents of change” [20] and include the beliefs, values, desires, and cognitive or emotional reasoning of participants and stakeholders who receive or deliver the intervention. They describe how the resources and activities embedded within an intervention influence the reasoning and ultimately the behavior of its participants [28]. The mechanisms are usually not directly observable or measurable [29, 30]. Outcomes are the expected or unexpected products of the interaction between mechanisms and context [27].

Realist reviews, as originally described by Pawson et al. [31], require considerable and sustained investment over time, which does not always suit the time-sensitive demands of many intervention or policy decisions. Recently, a ‘Rapid Realist Review’ methodology (RRR) has been developed as a tool for applying a realist approach to a knowledge synthesis process and producing a product that is useful in responding to time-sensitive issues with limited resources [32].

## Methods

### Approach

Utilizing the realist outline for systematic reviews [31], and adapting it to follow a RRR [32], the following steps will be applied:

#### 1. Clarifying the scope and articulating the preliminary program theory.

**Fig. 1:**
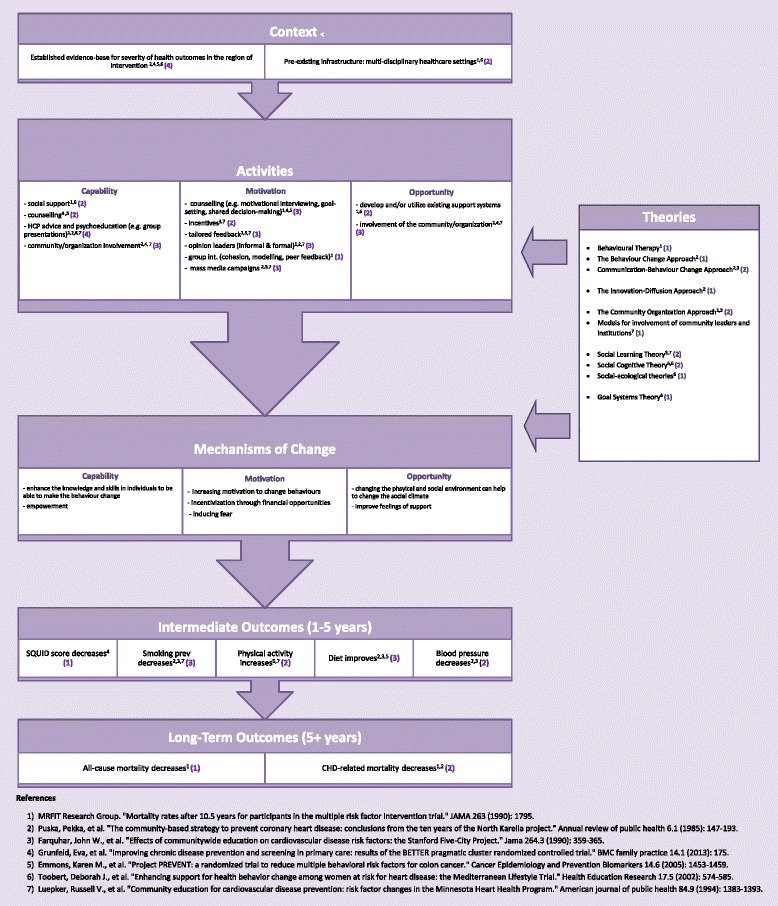
Preliminary Program Theory

We conducted a preliminary review to summarize the different possible theories and refine the research question. To determine the initial scope of this project, some of the published evidence from seven large-scale multi-factorial cardiovascular disease (CVD) and cancer risk factor interventions [16, 18, 21, 23, 33–35] were reviewed and the following data was extracted and summarized: study characteristics (year, population, sample size, setting, and type of intervention or program); design of the study; any explicit reference to a theory underlying the intervention; and any theoretical description linking context (C), mechanism (M) of change, and outcome (O) [27]. This dataset is available upon request. From this preliminary review, themes regarding the contexts, activities, mechanisms, and outcomes incorporated within effective multiple health behavior change (MHBC) interventions began to emerge. In some cases, the mechanisms of change were not explicitly mentioned but could be inferred from the article. Our expert panel reviewed the results of the mapping exercise and assisted the research team in developing the preliminary program theory using concepts from the Behavior Change Wheel [36], a theory-driven method for characterizing and designing behavior change interventions (Fig. 1). The expert panel had a total of 11 members and was comprised of representatives from the Centre for Addiction and Mental Health, Medical Psychiatry Alliance, and Public Health Ontario. They were engaged through in-person meetings and via email and were asked to share their expertise and provide feedback as it relates to chronic disease risk factors and integrated chronic disease prevention. The panel met 9 times in-person over a period of 6 months.

##### Context

Two important contextual factors arose from this preliminary review: the importance of infrastructure and determining the appropriate regions to target when implementing MHBC interventions.

Having an existing multi-disciplinary infrastructure (e.g., access to nurses, dietitians) that the MHBC intervention can leverage [15, 24, 26, 27] and a geographical region where population level data showed there was a health problem that could be improved with behavior changes [16, 23, 33, 35] were shown to be an important part of the intervention design.

##### Mechanism and key components of the interventions

Several studies targeted not only the individuals but also the surrounding community and/or organizational structures through the integration of healthcare services, involvement of community leaders and schools, and modeling by peers and opinion leaders [21, 23, 33, 37]. This multi-level approach to intervening within the community appears to be in an effort to change the physical and social opportunities that can help facilitate MHBC in individuals. The preliminary mechanisms of change could be that by intervening with the community, the intervention improves feelings of support by participants and changes the social climate regarding multiple health behaviors (MHBs). Moreover, a few MHBC programs were coupled with mass media campaigns such as education, risk-messaging, and study information [21, 33, 34]. The mechanism of change for these mass media interventions was to provoke resolution of cognitive dissonance by combining fear-inducing messages with clear attainable recommendations for how to change the risk behavior [33].

At the individual level, several mechanisms of change were identified. These included increasing participants awareness of the problem and how to change the behavior through educational group presentations and health practitioner advice [21, 23, 33, 35, 37], and increasing participants’ feeling of being supported [35, 37] and empowered through counseling techniques such as motivational interviewing, goal-setting, and shared decision making [21, 23, 37]. Providing financial incentives was another way some interventions encouraged behavior change [21, 34].

Another key emerging theme within these studies is that the intervention was delivered over several sessions [21, 33, 35, 37]. Of the large-scale interventions reviewed to date, the BETTER Trial was the only study in which the individual-level intervention was a single 1-h session [23]. For studies in which the intervention spanned several sessions, tailored messages and feedback appeared to be an important component in the monitoring and reinforcement of MHBC [16, 21, 37].

The design of these interventions was primarily guided by four theoretical frameworks including the social learning theory [21, 34], social cognitive theory [16, 35], theories for community organization [21, 33, 34], and a theory of behavior change [33, 37].

##### Outcomes

Since MHBC interventions involve the simultaneous or concurrent change of health behaviors, the effect of individual interventions on mortality and morbidity need to be measured long term. For example, the MRFIT was initially designated a failure after 7 years when it failed to show a reduction in CHD mortality rates in the intervention group [25, 38]. However, since the completion of the trial, the long-term efficacy of the MRFIT cohort has been assessed at multiple follow-up periods with numerous studies demonstrating a decrease in CHD mortality [18, 39–41]. Thus, when measuring the outcomes of an intervention, it is important to consider factors such as loss to follow-up, adherence to the program, and follow-up periods to accurately detect differences in outcomes. Furthermore, MHBC program outcomes have been traditionally reported on each individual health behavior separately, which may lead to a diversity of different outcomes that undermines the conceptualization of an overall impact [42]. In recent years, more studies that intervene on multiple health behaviors, such as the BETTER trial, adopt a composite outcome measure that conveys a summary of changes made in multiple domains [23].

Any important protocol amendments will be fully documented in the methods section of the final paper.

#### 2. Searching for relevant evidence.

To test the program theory identified in step one, a search strategy will be implemented to retrieve relevant data from both published and grey literature. The search strategy will be developed by the research team in collaboration with a medical librarian, who will execute the search in multiple databases. A draft of the search strategy for MEDLINE can be found in Additional file 1.

The initial search will aim to identify as many multiple health behavior interventions as is possible, allowing the team to most accurately identify the trends which will inform later search iterations. Due to variation in the ways in which researchers write about such interventions, the initial search strategy will use a systematic approach to cast a wide net. Later search iterations may not be systematic or comprehensive, as is the convention in realist reviews [24, 29] .

The following bibliographic databases will be searched for peer reviewed literature: MEDLINE; EMBASE; PsycINFO; Cumulative Index to Nursing and Allied Health Literature (CINAHL); The Cochrane Library; Web of Science; Social Sciences Abstracts; and Social Work Abstracts. The strategy will include database-appropriate subject headings, as well as “keywords” in natural language. Search terms will include the following: *alcohol*, *tobacco*, *sleep*, *physical activity*, *nutrition*, *stress*, *intervention*, *treatment*, *programs*, and related terms. Terms and concepts will be combined using Boolean logic and operators including proximity searching. The strategy will not be limited by study design, in keeping with standards for this review type [29]. Apart from published articles from the seven large-scale multi-factorial cardiovascular disease (CVD) and cancer risk factor interventions [16, 18, 21, 23, 33–35] we have started reviewing, the search will be limited to articles published in English, from January 2005 to the present.

To identify grey literature from Canada, Europe, and the USA not already captured, the following websites and online repositories will be searched: Health Canada; Canadian Public Policy Collection; Clinicaltrials.gov; Clinicaltrialsregister.eu; Grey Literature Report; the Campbell Collaboration Library; and DesLibris. Reference lists of chosen articles and documents will also be hand searched in order to identify any relevant resources not captured by the systematic searches.

The systematic review software DistillerSR [43] will be used to assess the relevance of each study by two independent reviewers using a pre-designed relevance screening form (Additional file 2). Any disagreements will be discussed by a third reviewer of the author’s team until a consensus is reached.

Following a realist approach, it is possible that after developing the C-M-O configurations, knowledge users and the research team may modify these criteria.

#### 3. Data extraction

As in most realist reviews, the data extraction phase will occur in an iterative manner, allowing for new mechanisms to emerge at any time. A pre-designed data extraction form (Additional file 3) will be used by two independent reviewers to extract relevant information for each study using DistillerSR [43]. Two reviewers will independently extract data from the included studies. Any discrepancies between the reviewers will be resolved by consensus or a third reviewer where applicable. If deemed necessary, articles may be analyzed in NVivo [44]. A spreadsheet will be maintained with all articles that are to be included. Two researchers will identify recurrent themes within mechanisms and group to ascertain emerging patterns of C-M-Os. The researchers will infer from the data in the included documents what might be functioning as mechanisms. They will identify recurrent patterns in the data that relate to these mechanisms and develop C-M-O configurations for them. The researchers will then look for data from across all included documents to confirm, dismiss, or refocus these CMO configurations. If the articles included provide a description of their participants, we will use the PROGRESS (Place of residence, Race/ethnicity/culture/language, Occupation, Gender/sex, Religion, Education, Socioeconomic status, and Social capital) framework to ensure that we are using an equity lens in our review process [30].

#### 4. Appraising the quality of the evidence

A combination of the Mixed Methods Appraisal Tool (MMAT) [45] and the Critical Appraisal Skills Programme (CASP) [46] will be used to appraise the articles generated through the search. The MMAT has been validated to concurrently appraise the methodological quality of qualitative, quantitative, and mixed methods studies [47]. It contains 19 methodological quality criteria to assess the quality of five different types of studies including qualitative research, randomized controlled trials, non-randomized studies, quantitative descriptive studies, and mixed methods studies. These criteria are scored on a nominal scale (Yes/No/Can’t Tell), and an overall quality score can be ascertained using this tool for each included study. Additional criteria from CASP were added to account for reporting and attrition bias, relevance and transferability, and ethical considerations. Given that an overall quality score may not be informative, we may also create a descriptive summary using MMAT and CASP criteria. Using the pre-designed quality assessment form (Additional file 3), two reviewers will appraise the quality of the evidence from each study independently using DistillerSR [43]. During this appraisal stage, conflicts between reviewers will be resolved by consensus or a third reviewer where applicable.

The overall quality of the evidence will be reported to understand the validity of the included studies as a whole. When drawing conclusions from the completed dataset, the reviewers will also briefly report on the quality of evidence of the particular studies where applicable.

#### 5. Data synthesis

Findings from the literature review will form the basis for producing a final report which will provide answers to the following questions: (1) which factors are effective in MHBC; (2) where are these interventions effective; and (3) for whom are these interventions effective. In addition, the study team will refine the program theory to advance our current understanding on how MHBC interventions work in different contexts.

By extracting the data we collect in DistillerSR, we will construct a matrix using Excel or NVIVO 11 to collate information for each study on the following (Additional file 4):

- Health behaviors the intervention attempted to change
- Whether they tried to change the behaviors simultaneously or sequentially
- Target population
- Activities conducted
- Context they operated in
- Mechanisms of change postulated or assumed by the study’s authors to explain the success or failure of the intervention
- Outcomes for each change behavior
- Quality assessment score

We will examine the interaction between context, mechanism, and outcome and see how these map to our initial program theory. Studies with high-quality assessment score will be used to confirm, dismiss, or refocus the C-M-O configuration. Studies with a low quality assessment score will only be used to confirm an established C-M-O configuration, but not to modify it. We will have several meetings with our expert panel to discuss preliminary analysis and conclusions; thus, the synthesis will be derived from a negotiation between experts and reviewers.

A draft report will be disseminated to expert panel participants for input, using the RAMESES (Realist and Meta-narrative Evidence Syntheses: Evolving Standards) publication standards [21]. Members of the expert panel include representatives from provincial and national organizations who are experts in tobacco cessation and/or chronic disease risk factors, patients, professional associations, and public health. The report will include suggestions and recommendations in accordance with the quality and quantity of available evidence with regard to:

What interventions should be implemented to reduce multiple health risk behaviors?

What settings are best suited to implement these interventions?

Which of these interventions will produce the biggest impact on smoking cessation rates and reducing other risk behaviors?

The realist review aims to follow internationally recognized procedures and will be reported in accordance with the PRISMA guidelines where applicable [48] as shown in the PRISMA-P checklist (Additional file 5).

#### 6. Knowledge translation—recommendations and a plan for dissemination

An advisory committee consisting of 23 members including decision makers, researchers, physicians, and patients will develop a knowledge translation (KT) plan, which will include an integrated and interactive approach to inform the development of a curriculum to train health care practitioners (HCP) in how to address MHBs and later on the implementation of a scalable MHBC program in primary care clinics. Although the KT plan will focus on primary care sites across Canada, we will also develop a dissemination plan to share the findings with communities, municipalities, and other sectors of government who are working on similar initiatives.

## Discussion

We developed a realist review protocol to understand the multi-level mechanisms that may drive the success or failure of programs designed to support MHBC. The goal of the realist review is to provide important information regarding the mechanisms and contexts that explain the success or failure of interventions. This will be used to develop an online training and guidance tool for implementation into primary care, which will help support the delivery of MHBC interventions to clients. However, several potential limitations merit emphasis. First, research studies are generally not generated to be read with a realist lens and it is therefore possible that many studies will not report contextual or implementation factors. Publication bias is also possible, in that only studies reporting successful interventions might be reported. Both these gaps might be addressed in a later stage, by contacting authors and experts; once the rapid review is finalized, and a more thorough review is done. The final limitation is achieving a balance between a comprehensive review and a timely one. Bi-weekly the researchers will meet with the expert panel, to facilitate quick decision making with regards to identifying C-M-O configurations and helping create effective and useful categories, given the data retrieved from the articles.

## Additional files

Additional file 1: Appendix 1- Search Strategy. (DOC 31 kb)

Additional file 2: Appendix 2 – Relevance Screening Form. (DOCX 15 kb)

Additional file 3: Appendix 3 - Quality Assessment and Data Extraction Form. (DOCX 46 kb)

Additional file 4: Excel spreadseet. (XLSX 21 kb)

Additional file 5: PRISMA-P 2015 Checklist. (DOCX 37 kb)

## Abbreviations

BETTER: Trial Building on Existing Tools to Improve Chronic Disease Prevention and Screening in Family Practice
*CAMH*: Centre for Addiction and Mental Health
*CASP*: Critical Appraisal Skills Programme
*CHD*: Coronary heart disease
*CINAHL*: Cumulative Index to Nursing and Allied Health Literature
*C-M-O*: Context-mechanism-outcome
*CVD*: Cardiovascular disease
*DARE*: Database of Abstracts of Reviews of Effects
*ERIC*: Educational Resources Information Centre
*HCP*: Health care practitioners
*MHB*: Multiple health behavior
*MHBC*: Multiple health behavior change
*MMAT*: The Mixed Methods Appraisal Tool
*MRFIT*: The Multiple Risk Factor Intervention Trial
*PROGRESS*: Place of residence, Race/ethnicity/culture/language, Occupation, Gender/sex, Religion, Education, Socioeconomic status, and Social capital
*RAMESES*: Realist and Meta-narrative Evidence Syntheses: Evolving Standards
*RRR*: Rapid realist review

## References

[1] Public Health Agency of Canada: How healthy are Canadians? A trend analysis of the health of Canadians from a healthy living and chronic disease perspective in: Public Health Agency of Canada. 2017. https://www.canada.ca/en/public-health/services/publications/healthy-living/how-healthy-canadians.html. Accessed 15 June 2017.

[2] Manuel DG, Perez R, Sanmartin C, Taljaard M, Hennessy D, Wilson K, et al. Measuring burden of unhealthy behaviours using a multivariable predictive approach: life expectancy lost in Canada attributable to smoking, alcohol, physical inactivity, and diet. PLoS Med. 2016; 10.1371/journal.pmed.1002082. 10.1371/journal.pmed.1002082 PMC4986987 27529741

[3] Krueger H, Andres EN, Koot JM, Reilly BD. The economic burden of cancers attributable to tobacco smoking, excess weight, alcohol use, and physical inactivity in Canada. Curr Oncol. 2016; 10.3747/co.23.2952. 10.3747/co.23.2952 PMC4974031 27536174

[4] Leatherdale ST, Rynard V. A cross-sectional examination of modifiable risk factors for chronic disease among a nationally representative sample of youth: are Canadian students graduating high school with a failing grade for health? BMC Public Health. 2013; 10.1186/1471-2458-13-569. 10.1186/1471-2458-13-569 PMC3751757 23758659

[5] deRuiter WK, Cairney J, Leatherdale S, Faulkner G. The period prevalence of risk behavior co-occurrence among Canadians. Prev Med. 2016; 10.1016/j.ypmed.2015.11.026. 10.1016/j.ypmed.2015.11.026 26658026

[6] Klein-Geltink JE, Choi BC, Fry RN (2006). Multiple exposures to smoking, alcohol, physical inactivity and overweight: Prevalences according to the Canadian Community Health Survey Cycle 1.1. Chronic Dis Can.

[7] Schuit AJ, van Loon AJ, Tijhuis M, Ocke M (2002). Clustering of lifestyle risk factors in a general adult population. Prev Med.

[8] Poortinga W (2007). The prevalence and clustering of four major lifestyle risk factors in an English adult population. Prev Med.

[9] Alamian A, Paradis G. Clustering of chronic disease behavioral risk factors in Canadian children and adolescents. Prev Med. 2009; 10.1016/j.ypmed.2009.02.015. 10.1016/j.ypmed.2009.02.015 19254742

[10] Butterfield RM, Park ER, Puleo E, Mertens A, Gritz ER, Li FP (2004). Multiple risk behaviors among smokers in the childhood cancer survivors study cohort. Psychooncology.

[11] Deruiter WK, Faulkner G, Cairney J, Veldhuizen S. Characteristics of physically active smokers and implications for harm reduction. Am J Public Health. 2008; 10.2105/AJPH.2007.120469. 10.2105/AJPH.2007.120469 PMC2374828 18381990

[12] deRuiter WK, Cairney J, Leatherdale ST, Faulkner GE. A longitudinal examination of the interrelationship of multiple health behaviors. Am J Prev Med. 2014; 10.1016/j.amepre.2014.04.019. 10.1016/j.amepre.2014.04.019 25145617

[13] Auer R, Vittinghoff E, Kiefe C, Reis JP, Rodondi N, Khodneva YA, et al. Change in physical activity after smoking cessation: the Coronary Artery Risk Development in Young Adults (CARDIA) study. Addiction. 2014; 10.1111/add.12561. 10.1111/add.12561 PMC4088346 24690003

[14] Correa-Fernandez V, Diaz-Toro EC, Reitzel LR, Guo L, Chen M, Li Y, et al. Combined treatment for at-risk drinking and smoking cessation among Puerto Ricans: a randomized clinical trial. Addict Behav. 2017; 10.1016/j.addbeh.2016.10.009. 10.1016/j.addbeh.2016.10.009 PMC5358923 27825036

[15] Horn K, Branstetter S, Zhang J, Jarrett T, Tompkins NO, Anesetti-Rothermel A, et al. Understanding physical activity outcomes as a function of teen smoking cessation. J Adolesc Health. 2013; 10.1016/j.jadohealth.2013.01.019. 10.1016/j.jadohealth.2013.01.019 23578440

[16] Emmons KM, McBride CM, Puleo E, Pollak KI, Clipp E, Kuntz K (2005). Project PREVENT: a randomized trial to reduce multiple behavioral risk factors for colon cancer. Cancer Epidemiol Biomark Prev.

[17] Puska P, Nissinen A, Salonen JT, Toumilehto J (1983). Ten years of the North Karelia Project: results with community-based prevention of coronary heart disease. Scand J Soc Med.

[18] Mortality rates after 10.5 years for participants in the Multiple Risk Factor Intervention Trial (1990). Findings related to a priori hypotheses of the trial. The Multiple Risk Factor Intervention Trial Research Group. JAMA.

[19] Elder JP, McGraw SA, Abrams DB, Ferreira A, Lasater TM, Longpre H (1986). Organizational and community approaches to community-wide prevention of heart disease: the first two years of the Pawtucket heart health program. Prev Med.

[20] Winkleby MA, Taylor CB, Jatulis D, Fortmann SP (1996). The long-term effects of a cardiovascular disease prevention trial: the Stanford Five-City Project. Am J Public Health.

[21] Luepker RV, Murray DM, Jacobs DR, Mittelmark MB, Bracht N, Carlaw R (1994). Community education for cardiovascular disease prevention: risk factor changes in the Minnesota Heart Health Program. Am J Public Health.

[22] Toobert DJ, Glasgow RE, Strycker LA, Barrera M Jr, Ritzwoller DP, Weidner G. Long-term effects of the Mediterranean lifestyle program: a randomized clinical trial for postmenopausal women with type 2 diabetes. Int J Behav Nutr Phys Act. 2007; 10.1186/1479-5868-4-1. 10.1186/1479-5868-4-1 PMC1783667 17229325

[23] Grunfeld E, Manca D, Moineddin R, Thorpe KE, Hoch JS, Campbell-Scherer D, et al. Improving chronic disease prevention and screening in primary care: results of the BETTER pragmatic cluster randomized controlled trial. BMC Fam Pract. 2013; 10.1186/1471-2296-14-175. 10.1186/1471-2296-14-175 PMC4225577 24252125

[24] Pawson R, Greenhalgh T, Harvey G, Walshe K (2005). Realist review—a new method of systematic review designed for complex policy interventions. J Health Serv Res Policy.

[25] University of Minnesota. Multiple Risk Factor Intervention Trial (MRFIT). http://www.epi.umn.edu/cvdepi/study-synopsis/multiple-risk-factor-intervention-trial-mrfit/ (2012). Accessed 14 June 2017.

[26] Pawson R (2006). Evidence-based policy. A realist perspective.

[27] Pawson R, Tilley N (1997). Realistic Evaluation.

[28] Jagosh J, Macaulay AC, Pluye P, Salsberg J, Bush PL, Henderson J, et al. Uncovering the benefits of participatory research: implications of a realist review for health research and practice. Milbank Q. 2012; 10.1111/j.1468-0009.2012.00665.x. 10.1111/j.1468-0009.2012.00665.x PMC3460206 22709390

[29] Wong G, Greenhalgh T, Westhorp G, Buckingham J, Pawson R. RAMESES publication standards: realist syntheses. BMC Med. 2013; 10.1186/1741-7015-11-21. 10.1186/1741-7015-11-21 PMC3558331 23360677

[30] Astbury B, Leeuw F. Unpacking black boxes: mechanisms and theory building in evaluation. Am J Eval. 2010; 10.1177/1098214010371972.

[31] Pawson R, Greenhalgh T, Harvey G, Walshe K (2005). Realist review: a new method of systematic review designed for complex policy interventions. J Health Serv Res Policy..

[32] Saul JE, Willis CD, Bitz J, Best A. A time-responsive tool for informing policy making: rapid realist review. Implement Sci. 2013; 10.1186/1748-5908-8-103. 10.1186/1748-5908-8-103 PMC3844485 24007206

[33] Puska P, Nissinen A, Tuomilehto J, Salonen JT, Koskela K, McAlister A (1985). The community-based strategy to prevent coronary heart disease: conclusions from the ten years of the North Karelia project. Ann Rev Public Health.

[34] Farquhar JW, Fortmann SP, Flora JA, Taylor CB, Haskell WL, Williams PT (1990). Effects of communitywide education on cardiovascular disease risk factors. The Stanford Five-City project. JAMA.

[35] Toobert DJ, Strycker LA, Glasgow RE, Barrera M, Bagdade JD (2002). Enhancing support for health behavior change among women at risk for heart disease: the Mediterranean Lifestyle Trial. Health Educ Res.

[36] Michie S, van Stralen M, West R. The behaviour change wheel: a new method for characterising and designing behaviour change interventions. Implement Sci. 2011; 10.1186/1748-5908-1186-1142. 10.1186/1748-5908-6-42 PMC3096582 21513547

[37] Multiple Risk Factor Intervention Trial Research Group (MRFIT). Mortality after 10 1/2 years for hypertensive participants in the Multiple Risk Factor Intervention Trial. Circulation. 1990;82:1616–28. 10.1161/01.cir.82.5.1616 2225366

[38] Multiple Risk Factor Intervention Trial Research Group (MRFIT). Coronary heart disease death, nonfatal acute myocardial infarction and other clinical outcomes in the Multiple Risk Factor Intervention Trial. Am J Cardiol 1986;58:1-13. 10.1016/0002-9149(86)90232-8 2873741

[39] Multiple risk factor intervention trial (1982). Risk factor changes and mortality results. Multiple risk factor intervention trial research group. JAMA.

[40] Multiple Risk Factor Intervention Trial Research Group (MRFIT). Mortality after 16 years for participants randomized to the Multiple Risk Factor Intervention Trial. Circulation. 1996;94:946–51. 10.1161/01.cir.94.5.946 8790030

[41] Stamler J, Neaton JD, Cohen JD, Cutler J, Eberly L, Grandits G, et al. Multiple risk factor intervention trial revisited: a new perspective based on nonfatal and fatal composite endpoints, coronary and cardiovascular, during the trial. J Am Heart Assoc. 2012; 10.1161/JAHA.112.003640. 10.1161/JAHA.112.003640 PMC3541632 23316301

[42] Prochaska JJ, Velicer WF, Nigg CR, Prochaska JO. Methods of quantifying change in multiple risk factor interventions. Prev Med. 2008; 10.1016/j.ypmed.2007.07.035. 10.1016/j.ypmed.2007.07.035 PMC2288581 18319099

[43] DistillerSR (2017). Evidence partners, Ottawa, Canada.

[44] QSR International. NVivo qualitative data analysis Software, QSR International Pty Ltd, Version 11, 2015.

[45] Pluye P, Hong QN. Combining the power of stories and the power of numbers: mixed methods research and mixed studies reviews. Ann Rev Public Health. 2014; 10.1146/annurev-publhealth-032013-182440. 10.1146/annurev-publhealth-032013-182440 24188053

[46] Critical Skills Appraisal Programme. http://www.casp-uk.net/casp-tools-checklists (2017). Accessed 28 June 2017.

[47] Pace R, Pluye P, Bartlett G, Macaulay AC, Salsberg J, Jagosh J, et al. Testing the reliability and efficiency of the pilot mixed methods appraisal tool (MMAT) for systematic mixed studies review. Int J Nurs Stud. 2012; 10.1016/j.ijnurstu.2011.07.002. 10.1016/j.ijnurstu.2011.07.002 21835406

[48] Moher D, Liberati A, Tetzlaff J, Altman DG. Preferred reporting items for systematic reviews and meta-analyses: the PRISMA statement. PLoS Med. 2010; 10.1371/journal.pmed.1000097. PMC3090117 21603045

