# Supplementary material for: The effects of interventions targeting multiple health behaviors on smoking cessation outcomes: a rapid realist review protocol
Source: Syst Rev. 2018 Mar 1;7:38. doi: 10.1186/s13643-018-0702-0 (PMC5831832; doi:10.1186/s13643-018-0702-0)
Supplement: Supplementary file 1 — Appendix 1- Search Strategy. (DOC 31 kb) [file 13643_2018_702_MOESM1_ESM.doc]

## APPENDIX 1: Search Strategy

Database: Ovid MEDLINE(R) <1946 to May Week 4 2017>

June5 2017

Search Strategy:

--------------------------------------------------------------------------------

1 "Tobacco Use Disorder"/ (10202)

2 "Tobacco Use"/ (806)

3 exp Smoking/ (138767)

4 ((Cigar* or tobacco or nicotine) adj5 (smoke* or smoking or user* or addict* or depend*)).ti,ab,kf,hw. (83712)

5 smoking.ti,ab,kf,hw. (229629)

6 smoking cessation/ (25104)

7 ((Cigar* or tobacco or nicotine) adj5 (cessation or quit* or abstain* or abstin* or reduc*)).ti,ab,kf,hw. (16926)

8 or/1-7 [smoking terms] (247147)

9 (multipl* or several or "more than two" or additional or integrat* or combin* or concurrent or cooccur* or co-occur* or multifactorial or multi-factorial).ti,ab,kf,hw. (4263298)

10 Health Behavior/ (43272)

11 risk reduction behavior/ (10410)

12 exp health promotion/ (67521)

13 life style/ (51274)

14 (health behavio?r* or health risk behavio?r*).ti,ab,kf,hw. (51747)

15 or/10-14 [health behaviour general] (159160)

16 9 and 15 ["multiple" words + health behaviour general] (33090)

17 ((multipl* or several or "more than two" or additional or integrat* or combin* or concurrent or cooccur* or co-occur* or multifactorial or multi-factorial) adj3 ((health* or risk* or lifestyle* or "life style*") adj3 (behavio?r* or factor*))).ti,ab,kf,hw. (20398)

18 ((multipl* or several or "more than two" or addition* or integrat* or combin* or concurrent or cooccur* or co-occur* or multifactorial or multi-factorial) adj3 ((behavio?r* or lifestyle* or "life style*") adj3 (change* or modif*))).ti,ab,kf,hw. (1369)

19 or/16-18 [all multiple health behaviour terms] (52283)

20 exp alcohol drinking/ (61155)

21 exp alcohol-related disorders/ (107089)

22 exp alcoholic intoxication/ (12078)

23 exp alcoholic beverages/ (17456)

24 exp drinking behavior/ (67354)

25 Alcohol*.ti,ab,kf,hw. (334060)

26 (Alcohol* adj3 (drink* or consum* or abus* or intake)).ti,ab,kf,hw. (102305)

27 "alcohol use".ti,ab,kf,hw. (23590)

28 ((Heavy or hazardous or binge or excess*) adj3 (drink* or alcohol*)).ti,ab,kf,hw. (17993)

29 (Alcohol* adj3 (abstain* or abstinen* or detox*)).ti,ab,kf,hw. (4383)

30 (Alcohol* adj3 treat*).ti,ab,kf,hw. (12649)

31 (beer or wine or cider or spirits or liquor).ti,ab,kf,hw. (26379)

32 or/20-31 [all alcohol terms] (361572)

33 exp Exercise/ (157411)

34 exercise therapy/ (33943)

35 physical fitness/ (25769)

36 (Physical* adj3 (activ* or inactiv* or fit or fitness or endur*)).ti,ab,kf,hw. (118809)

37 Exerci*.ti,ab,kf,hw. (294019)

38 (sport* or walk* or run* or jog* or bike* or biking or bicycl* or swim* or aerobic*).ti,ab,kf,hw. (415462)

39 Sedentar*.ti,ab,kf,hw. (23655)

40 or/33-39 [all physical activity words] (692078)

41 exp Diet/ (242846)

42 Food habits/ (71856)

43 feeding behavior/ (71856)

44 food preferences/ (12035)

45 nutrition therapy/ (1725)

46 diet therapy/ (10032)

47 Nutritio*.ti,ab,kf,hw. (304708)

48 (Health* adj3 (food* or diet* or eat*)).ti,ab,kf,hw. (22610)

49 (Unhealth* adj3 (food* or diet* or eat*)).ti,ab,kf,hw. (2384)

50 (Fruit* or Vegetable*).ti,ab,kf,hw. (107788)

51 ((food* or fat or fats or calori*) adj2 (intake or consum*)).ti,ab,kf,hw. (67413)

52 or/41-51 [all healthy eating words] (662289)

53 Stress, psychological/ (105270)

54 Relaxation therapy/ (6213)

55 ((emotion* or life or psychologic*) adj3 (stress* or suffer*)).ti,ab,kf,hw. (118766)

56 (Stress* adj3 manage*).ti,ab,kf,hw. (5219)

57 Relax*.ti,ab,kf,hw. (133546)

58 Mindful*.ti,ab,kf,hw. (4519)

59 Meditat*.ti,ab,kf,hw. (4408)

60 53 or 54 or 55 or 56 or 57 or 58 or 59 (258049)

61 (((posttraumatic or post-traumatic) adj1 stress*) or ptsd).ti,ab,kf,hw. (23815)

62 60 not 61 (254790)

63 stress test*.ti,ab,kf,hw. (11546)

64 62 not 63 [stress terms] (253426)

65 exp Sleep/ (70291)

66 exp Sleep Wake Disorders/ (75853)

67 sleep*.ti,ab,kf,hw. (155501)

68 insomnia*.ti,ab,kf,hw. (14568)

69 or/65-68 [sleep terms] (168126)

70 (program* or intervention* or treat* or modalit* or trial*).ti,ab,kf,hw. (6163693)

71 dt.fs. (2001952)

72 rh.fs. (183300)

73 th.fs. (1678968)

74 pc.fs. (1181599)

75 or/70-74 [intervention terms] (8156456)

76 8 and 75 [smoking and intervention] (107049)

77 76 and 19 [smoking + MHB + intervention] (4934)

78 76 and 32 and 40 [smoking + alcohol + exercise + intervention] (3871)

79 76 and 32 and 52 [smoking + alcohol + nutrition + intervention] (2936)

80 76 and 32 and 64 [smoking + alcohol + stress + intervention] (564)

81 76 and 32 and 69 [smoking + alcohol + sleep + intervention] (485)

82 76 and 40 and 52 [smoking + exercise + nutrition + intervention] (4112)

83 76 and 40 and 64 [smoking + exercise + stress + intervention] (616)

84 76 and 40 and 69 [smoking + exercise + sleep + intervention] (404)

85 76 and 52 and 64 [smoking + nutrition + stress + intervention] (387)

86 76 and 52 and 69 [smoking + nutrition + sleep + intervention] (198)

87 76 and 64 and 69 [smoking + stress + sleep + intervention] (107)

88 or/77-87 [all health behaviour intervention combos] (12183)

89 (editorial or letter).pt. (1339332)

90 88 not 89 (12119)

91 limit 90 to yr="2005 -Current" (7509)

92 limit 91 to english language (6902)

***************************
