# Supplementary material for: The effects of interventions targeting multiple health behaviors on smoking cessation outcomes: a rapid realist review protocol
Source: Syst Rev. 2018 Mar 1;7:38. doi: 10.1186/s13643-018-0702-0 (PMC5831832; doi:10.1186/s13643-018-0702-0)
Supplement: Supplementary file 2 — Appendix 2 – Relevance Screening Form. (DOCX 15 kb) [file 13643_2018_702_MOESM2_ESM.docx]

## APPENDIX 2: Relevance Screening Form

## What factors are associated with effective multiple health behaviour change (three or more, including smoking cessation)?

**Relevance Screening Form for Titles and Abstracts:**

| **Question** | **Options** | **Definitions/additional notes** |
| --- | --- | --- |
| Does this citation describe **interventions** that change **tobacco use** as well as **two or more** additional unhealthy **behaviours** (multiple health behaviour change)? | - Yes – relevant research - No - not relevant (excluded, submit form) | **Interventions:** a program or strategy designed to produce behaviour changes or improve health among individuals or populations. This can include educational programs, policies, health promotion campaigns etc.  **Behaviours:** including nutrition, alcohol consumption, physical activity, stress and mood, and sleep. |

2 reviewers independently will evaluate each citation.
